# Assessing immunogenicity of CRISPR-NCas9 engineered strain against porcine epidemic diarrhea virus

**DOI:** 10.1007/s00253-023-12989-0

**Published:** 2024-03-02

**Authors:** Fengsai Li, Haiyuan Zhao, Ling Sui, Fangjie Yin, Xinzi Liu, Guihai Guo, Jiaxuan Li, Yanping Jiang, Wen Cui, Zhifu Shan, Han Zhou, Li Wang, Xinyuan Qiao, Lijie Tang, Xiaona Wang, Yijing Li

**Affiliations:** 1 College of Veterinary Medicine, Northeast Agricultural University, Harbin, 150030 China; 2 Hebei Key Laboratory of Preventive Veterinary Medicine, College of Animal Science and Technology, Hebei Normal University of Science and Technology, Qinhuangdao, 066004 China; 3 Heilongjiang Key Laboratory for Animal Disease Control and Pharmaceutical Development, Harbin, 150030 China

**Keywords:** CRISPR-Cas9, Lactic acid bacteria, Genome expression, PEDV S1 gene, Oral vaccine

## Abstract

**Abstract:**

Porcine epidemic diarrhea (PED) caused by porcine epidemic diarrhea virus (PEDV), is an acute and highly infectious disease, resulting in substantial economic losses in the pig industry. Given that PEDV primarily infects the mucosal surfaces of the intestinal tract, it is crucial to improve the mucosal immunity to prevent viral invasion. Lactic acid bacteria (LAB) oral vaccines offer unique advantages and potential applications in combatting mucosal infectious diseases, making them an ideal approach for controlling PED outbreaks. However, traditional LAB oral vaccines use plasmids for exogenous protein expression and antibiotic genes as selection markers. Antibiotic genes can be diffused through transposition, transfer, or homologous recombination, resulting in the generation of drug-resistant strains. To overcome these issues, genome-editing technology has been developed to achieve gene expression in LAB genomes. In this study, we used the CRISPR-NCas9 system to integrate the PEDV S1 gene into the genome of alanine racemase-deficient *Lactobacillus paracasei* △*Alr HLJ-27* (*L. paracasei* △*Alr HLJ-27*) at the thymidylate synthase (thyA) site, generating a strain, *S1/*△*Alr HLJ-27*. We conducted immunization assays in mice and piglets to evaluate the level of immune response and evaluated its protective effect against PEDV through challenge tests in piglets. Oral administration of the strain *S1/*△*Alr HLJ-27* in mice and piglets elicited mucosal, humoral, and cellular immune responses. The strain also exhibited a certain level of resistance against PEDV infection in piglets. These results demonstrate the potential of *S1/*△*Alr HLJ-27* as an oral vaccine candidate for PEDV control.

**Key points:**

• *A strain S1/△Alr HLJ-27 was constructed as the candidate for an oral vaccine.*

• *Immunogenicity response and challenge test was carried out to analyze the ability of the strain.*

• *The strain S1/△Alr HLJ-27 could provide protection for piglets to a certain extent.*

## Introduction

Porcine epidemic diarrhea (PED) is an intestinal infectious disease caused by the porcine epidemic diarrhea virus (PEDV). It is characterized by vomiting, severe diarrhea, and high mortality in piglets (Sun et al. 2019). The PEDV S protein is the main envelope glycoprotein of PEDV and is composed of S1 and S2 domains. The S1 gene enables viral particles to invade the body and stimulate immune cells to produce neutralizing antibodies; therefore, it is an excellent antigen candidate for vaccine production (Sun et al. 2021). Several studies have used the S1 gene to develop vaccines against PEDV, with promising results (Wen et al. 2018; Egelkrout et al. 2020). PEDV primarily invades mucosal surfaces, especially the intestinal mucosal epithelial surfaces; thus, it is crucial to develop an oral mucosal vaccine that can induce effective mucosal immune responses (Zhang et al. 2017). Many studies have used lactic acid bacteria (LAB) as delivery vectors to produce oral mucosal vaccines against intestinal pathogens, owing to their safety, absence of side effects, and non-specific immune adjuvant effects. Furthermore, LAB have many advantages, such as enhancing the non-specific immune ability of the body, regulating the immune response by stimulating cytokine synthesis, reducing inflammatory response signals, regulating dendritic cell functions, and regulating secreted levels of SIgA (Hartanti 2010, Wang et al. 2016, Zhang et al. 2017). Among the LAB, *Lactobacillus paracasei* (*L. paracasei*) has great potential as an antigen delivery host. Studies have shown that *L. paracasei* can persist in intestinal and vaginal tissues, as well as reduce the presence of pathogenic *Escherichia coli* and *Shigella* in the human intestinal microflora (Zampieri et al. 2012). Muhammed et al. utilized *L. paracasei* (DUP-13076; LP), *Lactobacillus rhamnosus* (nrrlb442; LR), and *Lactobacillus delbrueckii* subsp. *bulgaricus* (NRRLB548; LD) to immunize chickens, and the results showed that all three strains could be well colonized in chickens, effectively reducing the adhesion and invasion ability of Salmonella in intestines and reduce the expression of Salmonella virulence genes (Muyyarikkandy et al. 2017). Zeng et al. used *L. paracasei* as oral carrier to deliver exendin-4 peptide and successfully realized the secretion expression of foreign protein, replacing expensive chemical synthesis and inconvenient injection administration (Zeng et al. 2016).

LAB employ plasmids as a medium to deliver foreign antigens and use antibiotic genes as selective markers (Wang et al. 2019). However, there are many defects associated with the plasmid expression system. The presence of antibiotic genes can be diffused through transposition, transfer, or homologous recombination during operation and use, resulting in the emergence of drug-resistant strains (Whittle et al. 2002). Replication of the plasmid via the rolling circle mechanism leads to the appearance of an unstable single-chain intermediate in the middle link, leading to deletions in the genetic process (Douglas and Klaenhammer 2011; Evdokimova et al. 2019). Although high copy number plasmids might offer certain advantages for antigen expression, plasmid instability and the antibiotic selective pressure make their application in animals challenging (Song et al. 2014b; Zhao et al. 2020). Researchers have shifted their focus to the LAB genome (Russell and Klaenhammer 2001; Yang et al. 2015). Integrating genes into chromosomes through gene-editing technology for expression is expected to eliminate the use of antibiotic markers and guarantee the stable inheritance of foreign genes.

As research progresses, clustered regularly interspaced short palindromic repeats (CRISPR) sequences, an adaptive immune response, have been effectively developed as a gene-editing technology to facilitate convenient and rapid genome manipulation processes (Seo et al. 2023). Researchers have successfully utilized the CRISPR/Cas system to knock out and insert genes in a variety of animals and bacteria (Agarwal and Gupta 2021). However, there have been limited studies on the application of the CRISPR/Cas9 system on LAB. This system cuts the genome to induce double-stranded DNA breaks (DSB), but it has been observed to exhibit high lethality in some bacteria (Ran et al. 2013; Xu et al. 2015). Therefore, gene knockout and insertion operations in the genome of LAB still rely on the classical homologous recombination and double-crossover strategy, which is rather laborious and time-consuming (Biswas et al. 1993, Song et al. 2014a, 2014b). Recently, researchers have optimized the CRISPR/Cas9 system to successfully perform genetic manipulation in LAB. In 2014, Oh et al. combined the CRISPR/Cas9 system with ssDNA recombination to successfully edit the genome of *Lactobacillus reuteri* (Oh and van Pijkeren 2014). Guo et al. utilized an ssDNA recombineering technique with a modified CRISPR-Cas9 counterselection to successfully knock out the UPP gene in *Lactococcus lactis* (Guo et al. 2019). Song et al. applied an optimized CRISPR-NCas9 system to cut single DNA strands to overcome bacterial cell death caused by DNA double-strand breaks (Song et al. 2017).

In the study, we used the CRISPR-NCas9 system to construct the gene-editing plasmid pLCNICK-S1, which was employed to generate an alanine-deficient *L. paracasei S1/*△*Alr HLJ-27* expressing the PEDV S1 gene in the genome of *L. paracasei* △*Alr HLJ-27*. Immunogenicity upon administration was analyzed based on the levels of anti-PEDV IgG and mucosal IgA antibody responses in mice and piglets. Additionally, a challenge test was performed to further explore the immune protection provided by the *S1/*△*Alr HLJ-27* strain in piglets.

## Materials and methods

### Strains, plasmids, and virus

Auxotrophic *L. paracasei* △*Alr HLJ-27* was constructed (Li et al. 2022) based on the wild strain *HLJ-27*, isolated from the intestine of large landrace piglets and cultured in de Man, Rogosa, and Sharpe (MRS) broth supplemented with an additional 200 μg/mL d-alanine for stationary culture. The stability of this strain was evaluated, and then subjected to the expression of the VP4 gene in a previous study (Li et al. 2022). The homology of the alr and thyA gene was more than 99% with the engineered strain *L. paracasei W56*. The plasmid pLCNICK (Song et al. 2017), including the thermosensitive replicon, NCas 9 protein, and sgRNA sequence, was generously provided by Yang Sheng (a researcher at the Institute of Plant Physiology and Ecology, Shanghai Institutes for Biological Sciences, Chinese Academy of Sciences). Plasmids pMD-19Ts-P776 (Yin et al. 2016), pMD-19Ts-RBS, and pMD-19Ts-S1 (Xiao et al. 2022) were preserved in our laboratory. The PEDV LJB2019 strain was isolated from clinical samples and stored at − 140 °C to maintain its virulence. This strain could be reasonably obtained from Dr. Xiaona Wang.

### Construction and identification of the mutant strain *S1*/△*AlrHLJ-27* expressing the PEDV S1 gene genomically

**Fig. 1:**
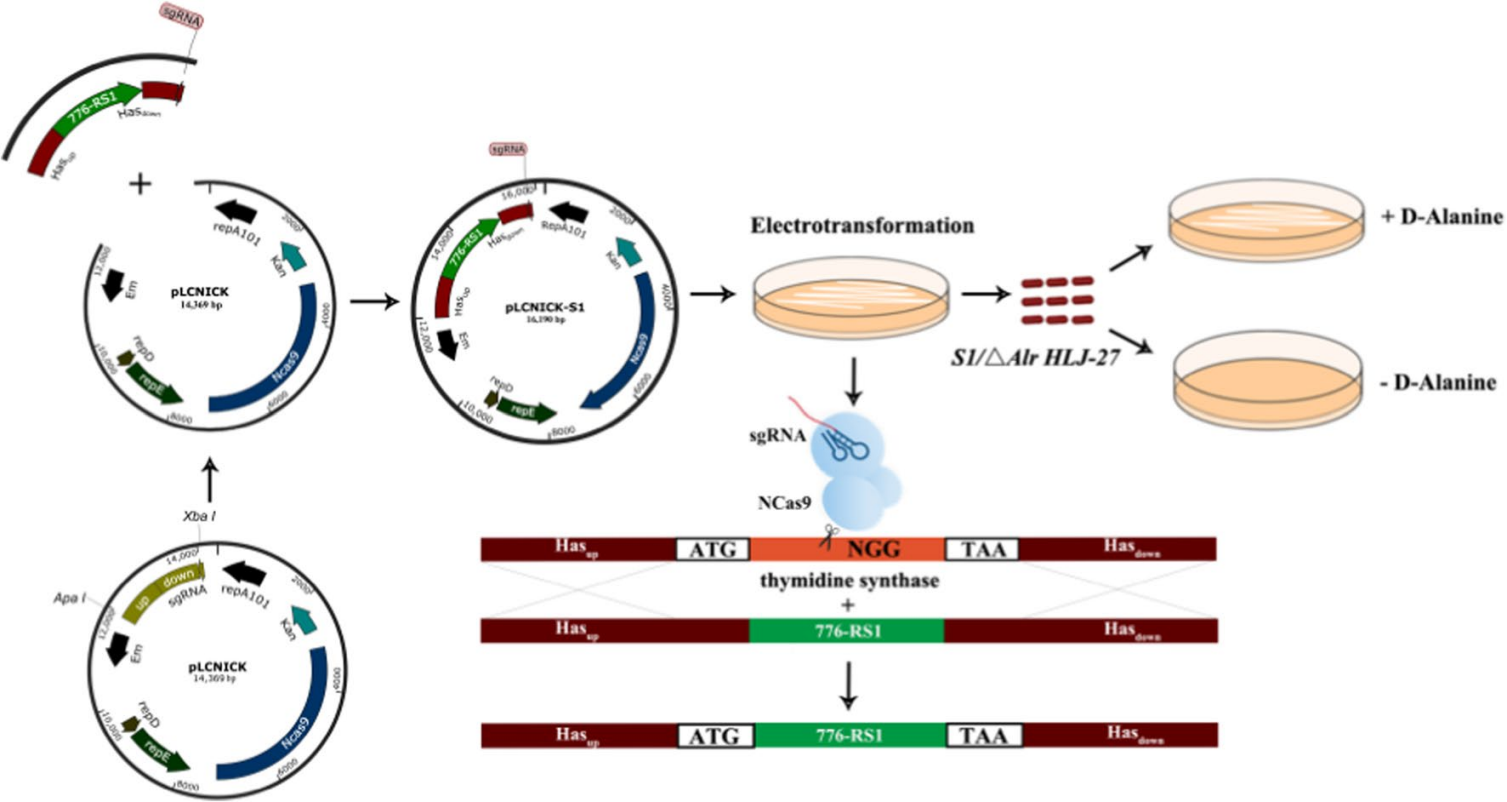
Schematic diagram of the construction of gene-editing plasmid pLCNICK-S1 and the gene-editing process CRISPR-Cas9^D10A^ system. The gene-editing plasmid pLCNICK-S1 was constructed as showing as the steps. The fragment P776 promoter, RBS, and S1 were purified and fused into one fragment, generating the gene P776-RBS-S1 (P776-RS1); the fragment Has_up_, P776-RS1, and Has_down_-sgRNA were amplified by the fusion PCR to yield the gene Has_up_-P776-RS1-Has_down_-sgRNA, then ligated with the pMD19T-Simple, producing the cloning plasmid pMD19Ts-Has_up_-P776-RS1-Has_down_-sgRNA (pMD19Ts-S1). The plasmid pMD19Ts-S1 was digested and then inserted into the temperature-sensitive vector pLCNICK at *Apa* I and *Xba* I sites, generating the gene-editing plasmid pLCNICK-S1. The pLCNICK-S1 was electrotransformed into competent cells △*Alr HLJ-27* to initiate the gene-editing process, yielding the mutant strain *S1*/△*Alr HLJ-27* with substituting the P776-RS1 gene for thymidine synthase (thyA) at the genome, and only growing with exogenous addition of d-alanine

The gene-editing plasmid construction strategy is shown in Fig. 1. The genes encoding the promoters P776, RBS, and S1 (the accession number, OR496609) were amplified using the standard PCR procedure with the plasmids pMD-19Ts-P776, pMD-19Ts-RBS, and pMD-19Ts-S1 as templates, respectively. The amplified fragments were then fused into one fragment, P776-RBS-S1 (P776-RS1), in the order of P776, RBS, and S1, via fusion PCR with the homologous sequence of the primers (P776-F, S1-R). The detailed primer sequences are listed in Table 1. The fragment Has_up_ and Has_down_-sgRNA were obtained from the genome of *L. paracasei HLJ-27* using PCR amplification (Has_up_-F/R and Has_down-_sgRNA-F/R as the primers). The fragment Has_up_-P776-RS1-Has_down_-sgRNA was then obtained by fusion PCR using the genes Has_up_, P776-RS1, and Has_down_-sgRNA as templates (Has_up_-F and Has_down_-sgRNA-R as the primers) and inserted into the vector pMD-19 T-simple, generating the plasmid pMD-19Ts-Has_up_-P776-RS1-Has_down_-sgRNA (pMD-19Ts-S1). pMD-19Ts-S1 and the gene-editing framework pLCNICK were digested using *Apa* I and *Xba* I enzymes, resulting in the recombinant plasmid pLCNICK-S1 with T4 ligase ligation. Plasmids were identified through sequential analysis.

**Table 1:** The sequence of primers

Genes	ID	Primers sequences
Has_up_	Has_up_-F	**tctttttctaaacta***gggccc*GGATCCCATTCAGATCGCCA
	Has_up_-R	tatgcTGTCTTCTTCCCTCCAGTGGG
P776	P776-F	**ggagggaagaagaca**GCATATTACAAAAAAGTCCTCTGCTC
	P776-R	**tatcgtcactcct**AAGGCACGTCCTTCTTTAATGG
RBS	RBS-F	**gtgcctt**AGGAGTGACGATAAAGATGAAATTAAA
	RBS-R	agcgaagcTCCATCAGCTTTAACTGTTGTGGC
S1	S1-F	GgaTGCATTGGTTATGCTGCCAA
	S1-R	gcttcgtCTACTTATCGTCGTCATCCTTGTAATCGTAAAAGAAACCAGGCAACT
Has_down_	Has_down_-F	**cgacgataagtag**ACGAAGCACATGCTTGGGC
	Has_down_-R	**aaggatgatatcacc***tctaga* GTTTTAGAGCTAGAAATAGC
thyA	thyA-F	CAAAGAAGAAAACAAAACTGAC
	thyA-R	ACGAACTACAAATGCACATAAC
pLCNICK	XA-F	CGAACCGTCTTATCTCCCAT
	XA-R	TTGCCTTTTCCGTCCAGAGC

Restriction enzyme recognition sites applied for cloning are shown in italics. Homologous sequence and sgRNA sequence were shown in bold and red words, respectively

To obtain the mutant strain *S1/*△*Alr HLJ-27*, △*Alr HLJ-27* competent cells were prepared and electroporated according to a previously described method (Wang et al. 2018). In brief, 1 mL of bacterial liquid was inoculated into 100 mL of MRS medium, which included 1% glycine and 200 μg/mL d-alanine, for static culturing at 37 °C until the OD_600_ reached 0.4 to 0.8. The cells were then pre-cooled on ice for 20 min and then harvested via centrifugation at 4000 rpm for 10 min. The resulting precipitate was washed and resuspended in 2 mL of ice-cold solution II containing 10% glycerol and 17% sucrose. Aliquots of 200 μL of competent cells were prepared in individual tube and preserved at 80 °C until further use. The gene-editing plasmid pLCNICK-S1 was prepared prior to electroporation. The plasmids were gently mixed with △*Alr HLJ-27* competent cells, and the mixture was transferred into a pre-cooled Gene Pulser (Bio-Rad, Hercules, CA, USA) disposable cuvette (inter-electrode distance of 0.4 cm), then subjected to a single electric pulse (2.2 V; 200 Ω; 25 µF) using the Gene Pulser (Bio-Rad). The electrotransformed strains were then cultured statically at 30 °C and identified through genome PCR using thyA-F/R as primers.

The process of genome manipulation using gene-editing plasmids in bacteria is shown in Fig. 1. To obtain the pure strain *S1/*Δ*Alr HLJ-27*, the Δ*Alr HLJ-27* strain containing the editing plasmid was streaked on MRS plates supplemented with 200 μg/mL d-alanine and cultured at 37 °C for three to five generations. The edited plasmid was removed during this process. Once the purified strain *S1/*△*Alr HLJ-27* was obtained, its requirement for d-alanine was verified. The strain was then inoculated into MRS broth medium supplemented with 200 μg/mL d-alanine, or basic MRS broth medium, or streaked on MRS solid medium supplemented with 200 μg/mL d-alanine or basic MRS medium to observe its growth. To test the genetic stability of the inserted gene S1, the mutant strain *S1/*△*Alr HLJ-27* was streaked on MRS plates supplemented with 200 μg/mL d-alanine and serially passaged for 20 generations. The genome was extracted every two generations, and PCR and sequencing were used for identification. To evaluate the plasmid elimination, plasmids from the mutant strain *S1/*△*Alr HLJ-27* of both F_1_ and F_20_ generations were extracted and identified using PCR amplification with XA-F/R as primer pairs.

### Detecting the expression of the S1 protein

The mutant strain *S1/*Δ*Alr HLJ-27* was cultured overnight in MRS medium supplemented with 200 μg/mL d-alanine, and the bacterial precipitate was collected by centrifugation at 10,000 g for 2 min. The precipitate was incubated with 1% lysozyme and sonicated for 12% sodium dodecyl sulfate–polyacrylamide gel electrophoresis (SDS-PAGE) detection and western blotting. SDS-PAGE was used to separate the proteins in the mixture, which were then transferred onto PVDF membranes (Millipore, Milford, MA, USA) using a mouse anti-S1 monoclonal antibody (mAb cell supernatant) prepared in our laboratory as the primary antibody (Xiao et al. 2022). Horseradish peroxidase (HRP)-conjugated goat anti-mouse IgG antibody (Sigma, Ronkonkoma, NY, USA) was used as the secondary antibody at a dilution of 1:5000. The results were evaluated using a chemiluminescent substrate reagent (Thermo Scientific, Durham, NC, USA) according to the manufacturer’s instructions. To determine the stability of S1 gene expression in the mutant strain *S1/*Δ*Alr HLJ-27*, protein samples for the western blot assay were obtained with every five generations of the strain within 20 generations. The strain Δ*Alr HLJ-27* was used as a negative control.

### Oral immunization procedure and sample collection

**Fig. 2:**
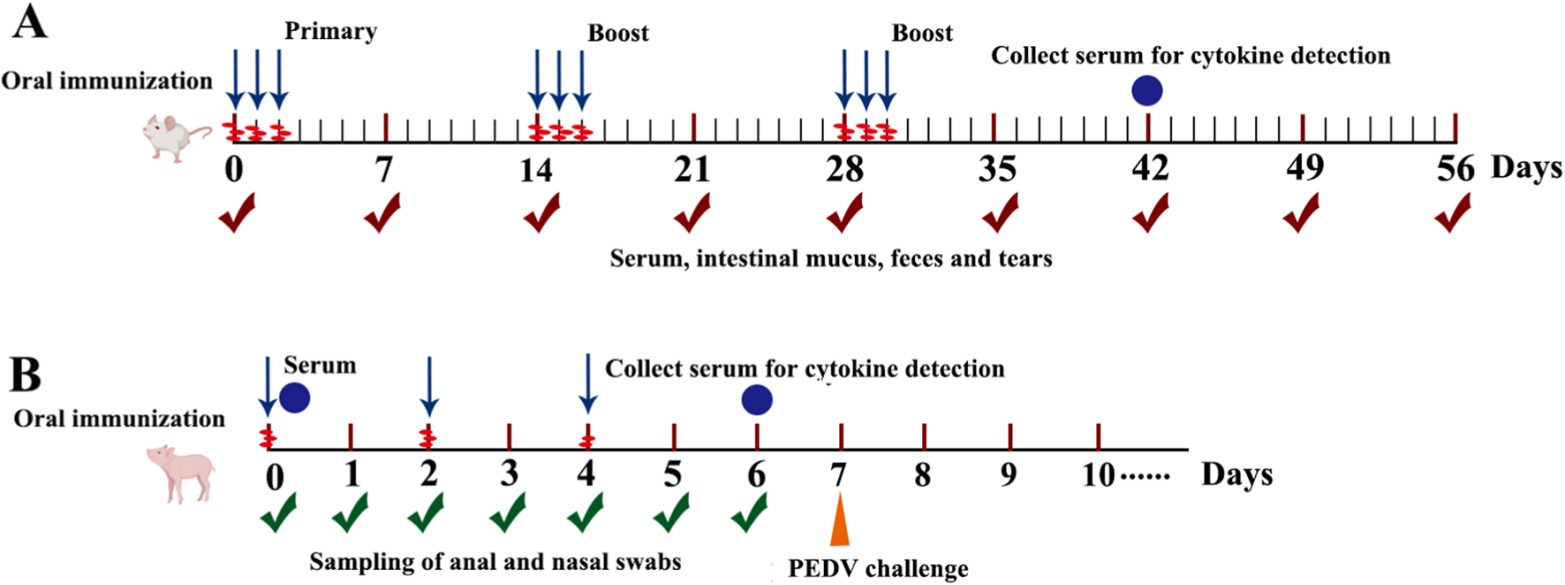
The procedure of mice (**A**) or piglets (**B**) with oral immunization and sampling. **A** Serum, intestinal mucus, feces, and tears were collected at the day of 7, 14, 21, 28, 35, 42, 49, and 56. The mice were 90 in total, for 30 mice per group, and each sampling was repeated three times (three mice). Cytokine detection was carried out on 42 days post primary immunization with gathering the serum from the eyeball. The immunization procedure adopts continuous immunization for the interval time with 2 weeks between twice booster immunization and each immunization continuously for 3 days; **B** Large landrace piglets were 15 in total, including *S1*/Δ*Alr HLJ-27* group (*n* = 6), Δ*Alr HLJ-27* group (*n* = 3) and PBS group (*n* = 6). Piglet serum was collected on days 0 and 6 post immunization. Anal and nasal swabs were collected daily and soaked in PBS. The piglet immunization procedure was immunized for 2 days at a time, with a total of three immunizations. The cytokine levels in sera were measured on day 6 post immunization and challenged the piglets with PEDV to determine the immune protection of mutant *S1*/△*Alr HLJ-27* with each group three piglets

All animal experimentation protocols were approved by the Institutional Committee of the Northeast Agricultural University for Animal Experiments (2016NEFU-315; April 13, 2017) in Harbin, China. Four-week-old female specific pathogen-free (SPF) BALB/c mice (*n* = 90, 30 per group) were obtained from Liaoning Changsheng Biotechnology Co., Ltd. (Liaoning, China). The mutant strain *S1/*Δ*Alr HLJ-27* was cultured overnight for 16 h, and the cells were then harvested by centrifugation to adjust the concentration to 1 × 10^10^ CFU/mL. The mice in the experimental group were orally administered 200 µL of the mutant strain *S1/*Δ*Alr HLJ-27*. Meanwhile, the other two groups of mice were orally administered 200 µL of Δ*Alr HLJ-27* and PBS as control groups, respectively. The procedure is shown in Fig. 2A.

Mice were orally immunized once every 14 days for a total of three times, consecutively for 3 days each time. Serum, feces, intestinal mucus, genital tract rinse, and tears were collected every 7 days for a duration of 56 days following the initial immunization. All samples were collected and stored at − 40 °C until further use. Serum was collected from the eyeball and diluted with 5% skim milk. The mice were then euthanized by cervical dislocation, and 0.5 g of intestinal mucus was scraped, mixed with 500 µL of PBS, and subjected to shaking and centrifugation to obtain the supernatant, which served as the primary antibody. Fecal pellets weighing 0.1 g were treated with 500-µL PBS containing 0.05 mmol EDTANa_2_ and lysed for 16 h at 4 °C to obtain the supernatant for detection. The genital tract and the tears were acquired with 500 µL PBS washing mouse vagina or eyes, respectively, then to used directly as primary antibodies.

Large landrace piglets 0 days after birth, which had not been vaccinated with the PEDV vaccine, were purchased from the A’cheng Experimental Practice Base of Northeast Agricultural University. These piglets were kept in an isolation device and provided with fresh water and sterile milk. A total of 15 piglets were randomly divided into three groups; the piglets were orally immunized with 2 mL of 1 × 10^10^ CFU/mL of the mutant strain *S1/*Δ*Alr HLJ-27* (*n* = 6), 2 mL of 1 × 10^10^ CFU/mL of Δ*Alr HLJ-27* (*n* = 3) and 2 mL of PBS (*n* = 6), respectively, using the same strain treatment method employed in mice. The immunization protocol for the piglets was based on previous research in piglets, with slight optimizations. The neonatal piglets received three immunizations at 0, 2, and 4 days, respectively (Fig. 2B). Serum was collected from the anterior vena cava of the piglets on days 0 and 6. From days 0 to 6, anal and nasal swabs were collected and soaked in 1 mL of PBS, and the supernatant was obtained through centrifugation and used as the primary antibody to detect specific SIgA levels. The samples were collected and preserved at − 80 °C until further analysis.

Six days after piglet immunization, a challenge test was performed. The piglets in the *S1/*Δ*Alr HLJ-27* (*n* = 3), Δ*Alr HLJ-27* (*n* = 3), and PBS (*n* = 3) groups were challenged with 1 mL of PEDV LJB2019 small intestinal tissue ground sample (1 mL of PEDV LJB2019 small intestinal tissue ground sample) using oral-gastric gavage. The remaining piglets in the PBS group (*n* = 3) served as negative controls, while the piglets in the *S1/*Δ*Alr HLJ-27* group (*n* = 3) were co-housed in the same cage with these challenge groups. Weight changes and the mental state of piglets were monitored daily. Piglets on the verge of death were immediately subjected to necropsy to analyze the protective effects provided by the mutant strain *S1/*Δ*Alr HLJ-27*.

### Analysis of IgG and SIgA levels

To detect the levels of IgG and SIgA antibodies, serum and mucosal samples were collected at corresponding time points and determined using the indirect ELISA method. The PEDV LJB2019 strain was coated on 96 polystyrene microtiter plates as the antigen, and then washed thrice with PBS containing 0.1% Tween 20 (PBST), blocked with 5% skim milk, and incubated at 37 °C for 2 h. After washing, the samples were added to the microplate with three repetitions and incubated at 37 °C for 2 h, serving as the primary antibody, while (HRP)-conjugated goat anti-mouse/pig IgG or IgA served as the secondary antibody and was incubated for 1 h. The TMB chromogenic solution (Sigma, Ronkonkoma, NY, USA) was mixed with A + B in equal proportions for visual detection at an absorbance of 490 nm.

To assess the neutralizing capacity of antibodies induced by the mutant strain *S1*/Δ*Alr HLJ-2*7 against virions, the levels of antibody neutralization in mouse or piglet serum were measured while maintaining a constant virus concentration. Serum samples (50 µL) were serially diluted by twofold and mixed with 50 µL of PEDV with a viral titer of 100 50% tissue culture infectious dose (TCID_50_); the mixture was then incubated at 37 °C for 1 h. Subsequently, 100 µL of the mixture, with eight repetitions, was added to vero cell monolayers, which had been grown to a density of 80% and incubated for 1 h. Next, 100 µL of maintenance solution was added to each well, and the plate was placed in a 5% CO_2_ 37 °C incubator for 72 h to observe cytopathic effects (CPE). Statistical analysis of the experimental results was performed using the Reed-Muench method.

### Cytokine assay

On day 42 after immunization, two mice from each group were euthanized to collect serum from the eyeball, and ELISA kits were used to measure cytokine levels (BioSource International, USA). Piglet serum was also assessed on day 6 after the initial immunization. Each sample was replicated three times, and blank wells were used as controls. The detected cytokines included interleukin-2 (IL-2), IL-4, IL-10, IL-12, IL-17, and interferon-γ (IFN-γ), and the cytokine concentrations were analyzed by drawing a standard curve to calculate the numerical values for each ELISA plate. After euthanization, piglet serum was collected to assess the inflammatory factors, including IL-6, IL-1β, and IL-10.

### qRT-PCR analysis

Real-Time quantitative RT-PCR (qRT-PCR) was used to determine the viral load in intestinal tissues using the ABI Prism 7500 sequence detection system (Applied Biosystems, Foster City, CA, USA) with SYBR green fluorescent dye. Prior to the assay, a standard curve was established for the absolute quantification of the virus. A standard plasmid with an initial concentration of 1 × 10^10^ copies/μL was subjected to a tenfold dilution, and each dilution was repeated five times for qRT-PCR. Using the logarithm of the dilution of the plasmid standard as the *x*-axis and the corresponding Ct value (cycle threshold) as the *y*-axis, a quantitative standard curve corresponding to the plasmid copy number and Ct value was constructed. Total RNA was extracted from 0.1 g of intestine samples and reverse-transcribed into complementary DNA (cDNA) according to the manufacturer’s instructions. The cDNA was diluted to the same level and prepared for qRT-PCR using a SYBR® qPCR Mix Reagent Kit (Takara) to test the PEDV copy number in the intestine samples.

### Gross lesion and histopathological examinations

Small intestinal tissues from the area with significant jejunal lesions were subjected to histopathological examination. Tissue samples were fixed in 4% polyformaldehyde, embedded into paraffin blocks, and sectioned at 3 µm. Hematoxylin–eosin (H&E) staining was utilized to observe the morphological changes of intestinal villi under a light microscope.

### Statistical analysis

The statistics were presented as mean ± SE of three replicates per test in an independent experiment. GraphPad Prism 8 and Excel 2010 were used to analyze data discrepancies. The significance between the experimental and control groups was determined using two-way and one-way analysis of variance, along with Tukey’s multiple-comparison test. * represents *p* < 0.05, indicating significance, while ** represents *p* < 0.01, indicating high significance.

## Results

### Construction and identification of the mutant strain *S1*/△*AlrHLJ-27*

**Fig. 3:**
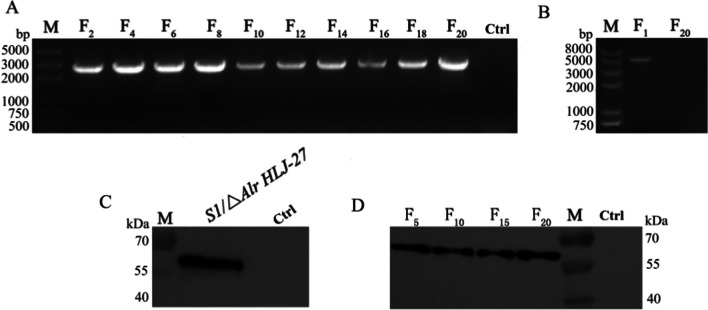
The gene hereditary, the plasmid elimination, and the stability of protein expression were detected of mutant strain *S1/*△*Alr HLJ-27* (**A**, **B**, **C**). The genome of the strain was extracted every two generation within 20 generations to determine the stability of the gene inheritance by PCR amplification with thy-F/R as the primers and sequencing (**A**), M: Trans 2 k plus DNA marker; F_2_-F_20_: The F_2_-F_20_ generation strains of *S1/*△*Alr HLJ-27*; Ctrl: the strain of △*Alr HLJ-27*. The plasmid elimination situation of F_1_ and F_20_ generation strains was evaluated by plasmid PCR amplification with XA-F/R as the primers and sequencing (**B**), M: Trans 2 k plus II DNA marker; F_1_/F_20_: The F_1_/F_20_ generation strain of *S1/*△*Alr HLJ-27*. The expression of interest protein was detected by western blotting with mouse anti-S1 monoclonal antibody as a primary antibody (**C**). The stability of protein expression every five generation was determined (**D**)

**Fig. 4:**
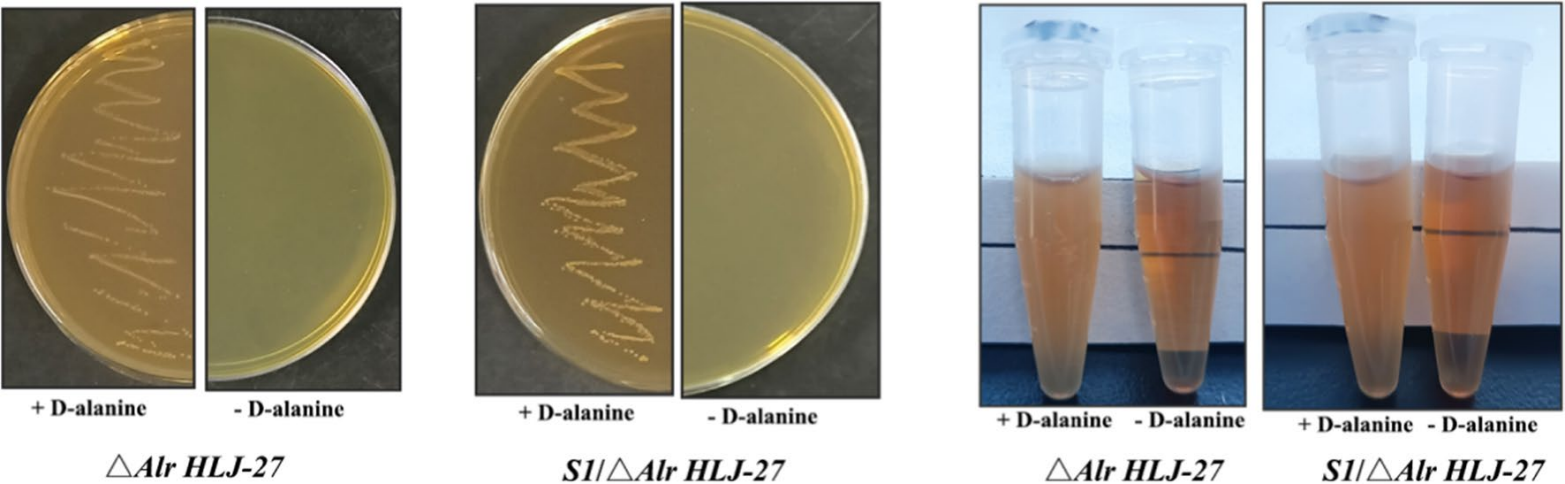
The demand test of the mutant strain of *S1/*△*Alr HLJ-27*. The growth results as the strain streaking on the MRS plate (**A**) and in the MRS liquid (**B**). “ + ” represent the MRS with the d-alanine; “ − ” represent the MRS without the d-alanine

The plasmid pLCNICK-S1 was electroporated into *L. paracasei* △*Alr HLJ-27* competent cells to generate the mutant strain *S1*/△*Alr HLJ-27*, in which the thymidylate synthase (thyA) gene was replaced by the P776-RS1 gene in the LAB genome. Genetic stability and plasmid elimination in strain *S1/*△*Alr* *HLJ-27* were detected. The strain was streaked on MRS plates containing 200 μg/mL d-alanine for 20 continuous passages, and genomic DNA was extracted every two generations for PCR identification. As shown in Fig. 3A, agarose gel electrophoresis showed that the genomic PCR amplification of the F_1_-F_20_ generation strains was 3415 bp bands, and the sequencing results confirmed the genetic stability of the locus, with no reverse mutation. Considering the passive effects of resistance gene residues in animals and humans, we conducted a PCR amplification test to examine the elimination of the gene-editing plasmid pLCNICK-S1, as shown in Fig. 3B. A 3956 bp band was observed in the F_1_ generation strain, in line with the expected size. Conversely, no band appeared in the F_20_ generation strain, indicating that the plasmid content decreased to undetectable levels or was completely eliminated. Based on the characteristics of the strain △*Alr HLJ-27*, the d-alanine requirement of the mutant strain *S1/*△Alr *HLJ-27* was tested. The strain was streaked on MRS plates containing 200 μg/mL d-alanine or basic MRS medium or inoculated in MRS broth supplemented with 200 μg/mL d-alanine or basic MRS medium for culturing. The results showed that the strain *S1/*△Alr *HLJ-27* could grow only when d-alanine was added, in line with its auxotrophic characteristics (Fig. 4).

### Expression of the target protein of S1 gene in strain *S1*/△*AlrHLJ-27*

The *S1/*△*Alr HLJ-27* strain was inoculated into MRS medium supplemented with 200 μg/mL d-alanine and cultured at 37 °C for 16 h. Subsequently, the cells were harvested using lysozyme and ultrasonication to analyze the expression of the S1 protein through western blotting. Mouse anti-S1 monoclonal antibody was used as the primary antibody. As shown in Fig. 3C, a specific immunoreactive band of about 60 kDa was observed in the lysate of the mutant strain *S1/*△*Alr HLJ-27*, as expected, but not in △*Alr HLJ-27*, showing that S1 was expressed successfully by strain *S1/*△*Alr HLJ-27*. Moreover, to test the stability of S1 protein expression in the *S1/*△*Alr HLJ-27* strain within 20 generations, protein samples were analyzed by western blotting every five generations (Fig. 3D). A specific band was detected in each generation of the strain, indicating the stable expression of the inserted foreign gene S1 in the mutant strain *S1/*△*Alr HLJ-27*.

### Detection of anti-PEDV IgG and SIgA levels, and analysis of PEDV-neutralizing activity of antibodies induced by the mutant strain *S*1/△*AlrHLJ-27* via oral immunization in mice

**Fig. 5:**
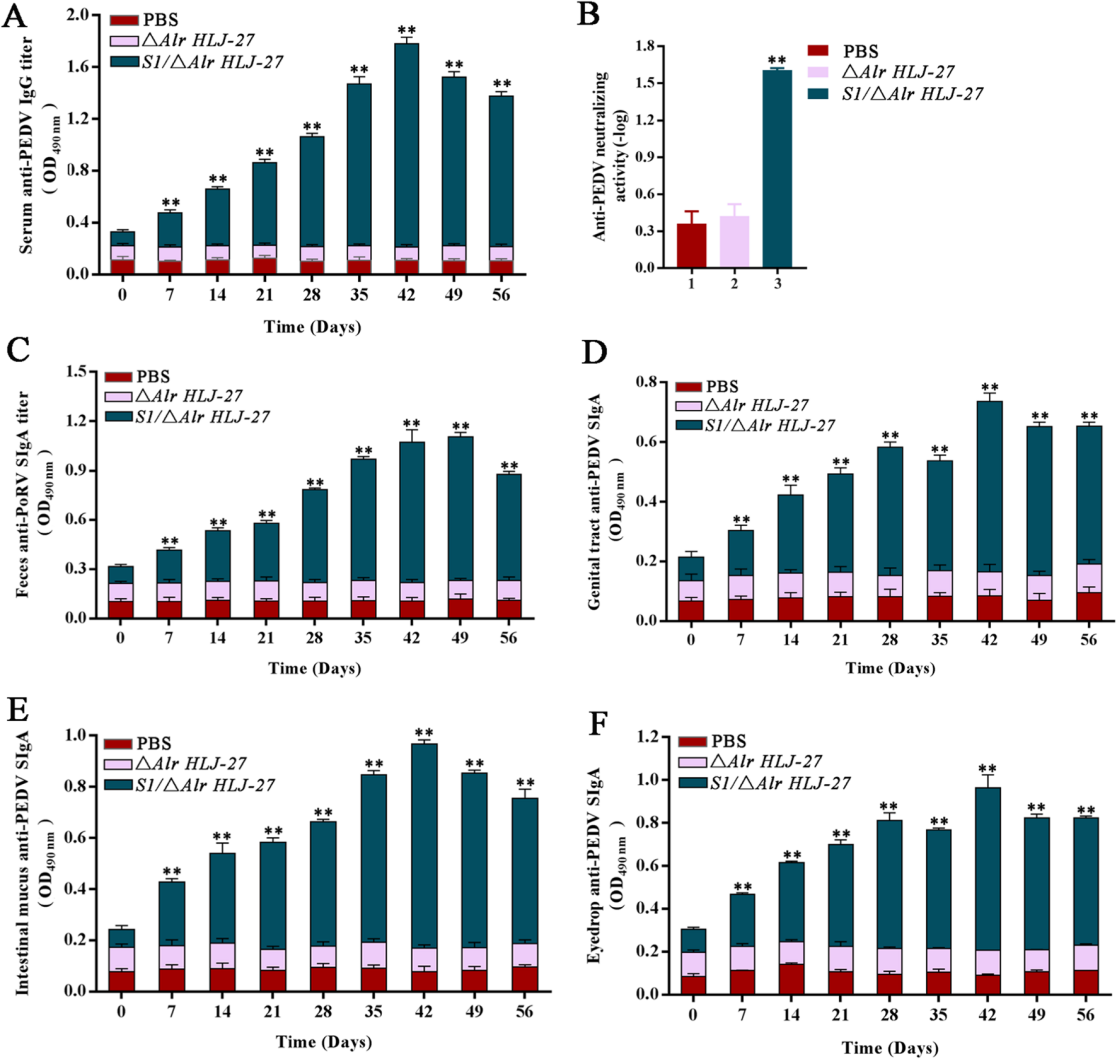
Determination of anti-PEDV levels of immunoglobulin G (IgG) (**A**) and secreted immunoglobulin A (SIgA) (**C**–**F**) in mice (*n* = 3 per group) with oral immunization the mutant strain *S1/*△*Alr HLJ-27*. Meanwhile, the neutralizing antibody activity of serum IgG was measured by the method for immobilizing viral load and to dilute antibodies (**B**). The supernatant of fecal lysis and intestinal mucus by centrifugation, eyedrop, genital tract rinse, and serum diluting with 5% skim milk were used as the primary antibodies. Bars represent the mean ± standard error value of each group (**p < 0.01 compared to the control groups PBS and △*Alr HLJ-27*)

To determine IgG and SIgA levels induced by the strain *S1/*△*Alr HLJ-27*, serum and the mucosal samples, including feces, intestinal mucus, genital tract rinse, and tears, were collected on days 0, 7, 14, 21, 28, 35, 42, 49, and 56 after the initial immunization and determined using indirect ELISA. Seven days after the initial vaccination, IgG (Fig. 5A) and SIgA (Fig. 5C–F) levels induced by the mutant strain increased gradually, reaching a peak at 42 days, and then started to decrease. During the entire process of monitoring antibody levels, the levels of IgG and SIgA produced by strain *S1/*△*Alr HLJ-27* were significantly higher than those in the △*Alr HLJ-27* and PBS groups. Two booster immunizations were administered on days 14 and 28. Subsequently, there were significant changes in the SIgA or IgG levels in the mutant strain *S1/*△*Alr HLJ-27* group.

The anti-PEDV-neutralizing activity induced by the mutant strain *S1/*△*Alr HLJ-27* was assessed while maintaining the number of viral particles (Fig. 5B). Compared to the △*Alr HLJ-27* group (1:3) and the PBS group (1:2), the level of neutralizing antibodies produced in the serum of mice stimulated by the mutant bacteria group *S1/*△*Alr HLJ-27* (1:40) was significantly increased. As expected, the strain effectively elicited an immune response against PEDV in vivo.

### Determination of cytokine responses in mice

**Fig. 6:**
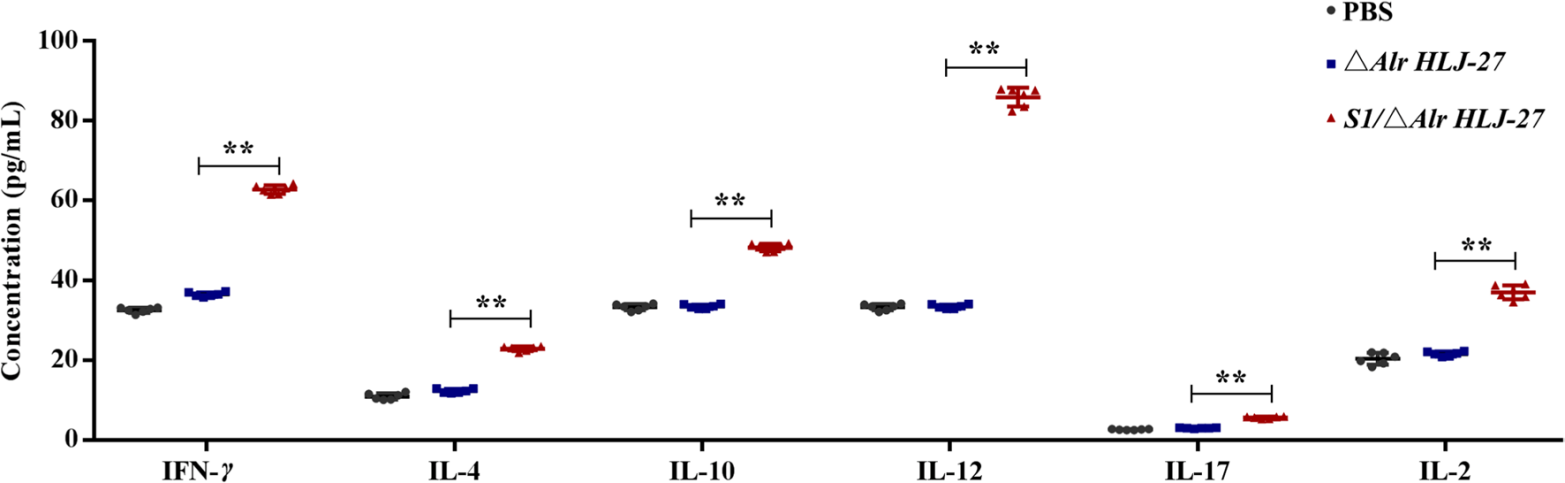
Detection of the levels of serum cytokines. The cytokines from the mice (*n* = 3 per group) were analyzed with oral immunization of the mutant strain *S1/*△*Alr HLJ-27*. Bars represent the mean ± standard error value of each group (**p < 0.01 compared to the control groups PBS and △*Alr HLJ-27*)

Fourteen days after the secondary booster immunization, serum was collected from the eyeball from two mice per group to assess the cytokine levels induced by *S1/*△*Alr HLJ-27*, as shown in Fig. 6. Based on the serum test, there was a significantly increase in IL-2, IL-4, IL-10, IL-12, IL-17, and IFN-γ level in the mutant *S1/*△*Alr HLJ-27* group compared with the △*Alr HLJ-27* and PBS groups.

### Detection of anti-PEDV IgG and SIgA levels and analysis of PEDV-neutralizing activity of antibodies induced by the mutant strain *S1*/△*AlrHLJ-27* via oral immunization in piglets

**Fig. 7:**
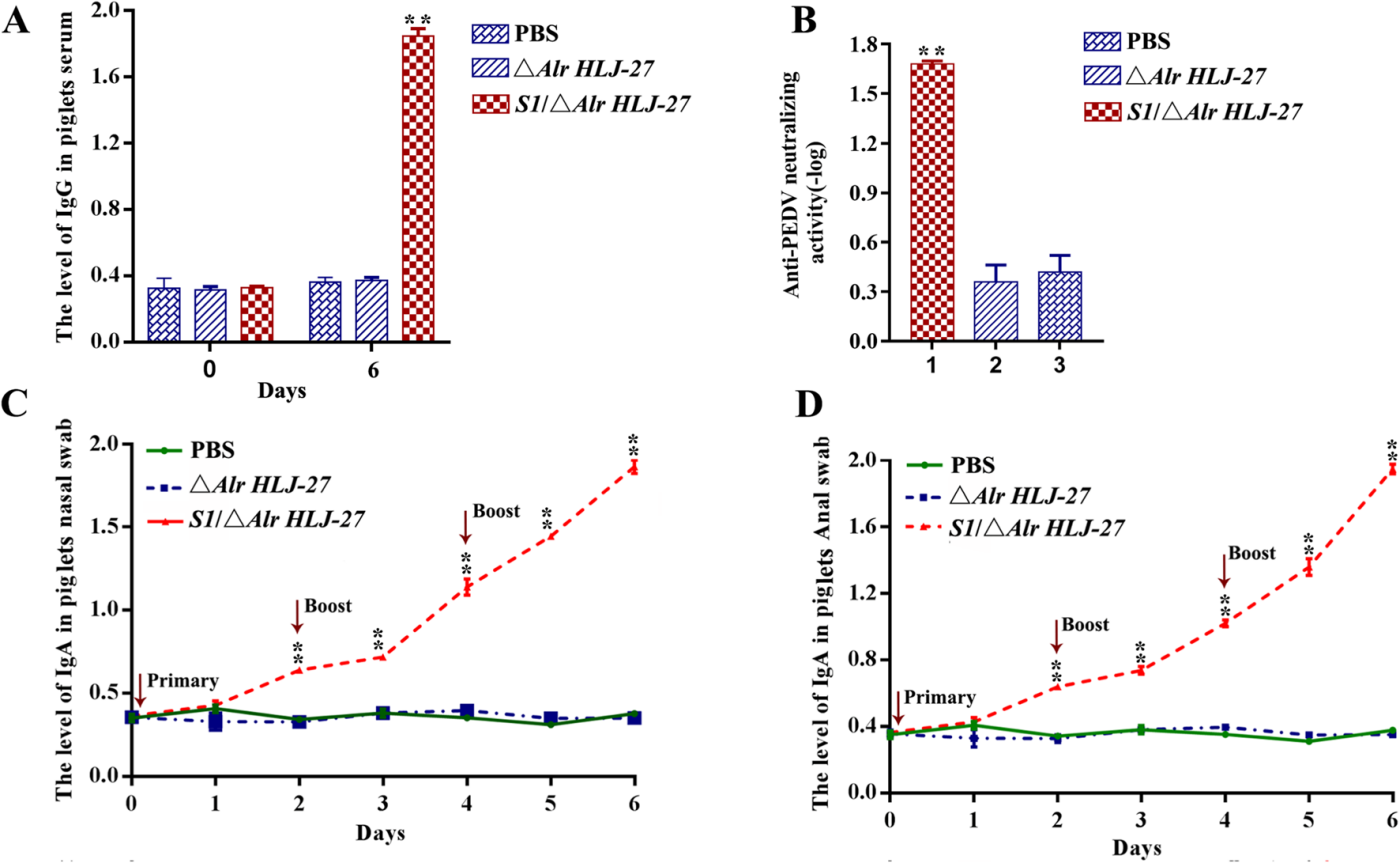
Determination of anti-PEDV levels of immunoglobulin G (IgG) (**A**) and secreted immunoglobulin A (SIgA) (**C**–**D**) in piglets (*n* = 3 per group) with oral immunization the mutant strain *S1/*△*Alr HLJ-27*. Meanwhile, the neutralizing antibody activity of serum IgG was measured by the method for immobilizing viral load and to dilute antibodies (**B**). The supernatant of anal and nasal swabs by centrifugation and serum diluting with 5% skim milk was used as the primary antibodies to detect the levels of SIgA and IgG. Bars represent the mean ± standard error value of each group (**p < 0.01 compared to the control groups PBS and △*Alr HLJ-27*)

To measure the SIgA antibody levels in piglets stimulated by *S1/*△*Alr HLJ-27*, anal and nasal swabs were collected from 0 to 6 days, and the supernatant was obtained through centrifugation for detection. The results are shown in Fig. 7C–D. SIgA levels in anal and nasal swabs gradually increased with the number of days of immunization. On day 6 after the primary immunization, the group receiving the strain *S1/*△*Alr HLJ-27* exhibited a significant increase in specific SIgA compared to the control groups. Following two booster immunizations, SIgA levels increased significantly, whereas before immunization, there was no significant statistical change in any of the groups. These results demonstrate that oral immunization with strain *S1/*△*Alr HLJ-27* induces a mucosal immunity response in piglets.

Serum was collected from the piglets via the anterior vena cava on days 0 and 6 to detect IgG antibody levels (Fig. 7A). Previously, there was no discrepancy in data between the △*Alr HLJ-27* and PBS groups and test group *S1/*△*Alr HLJ-27*. On day 6, the IgG level in the *S1/*△*Alr HLJ-27* group was markedly higher than that of the control group, indicating that the mutant strain could induce humoral immune response in piglets. These results show that antibodies induced by oral administration of the mutant strain in piglets could resist not only the virus particles in the gut but also the virus in vivo. The neutralizing activity in the serum induced by strain *S1/*△*Alr HLJ-27* in piglets was detected to evaluate the ability of the antibody to neutralize viruses (Fig. 7B). The level of neutralizing antibodies clearly increased in the *S1/*△*Alr HLJ-27* group (1:64) compared with that in the △*Alr HLJ-27* group (1:3) and the PBS group (1:2), indicating that *S1/*△*Alr HLJ-27* could activate an effective immune response.

### Determination of cytokine responses in piglets

**Fig. 8:**
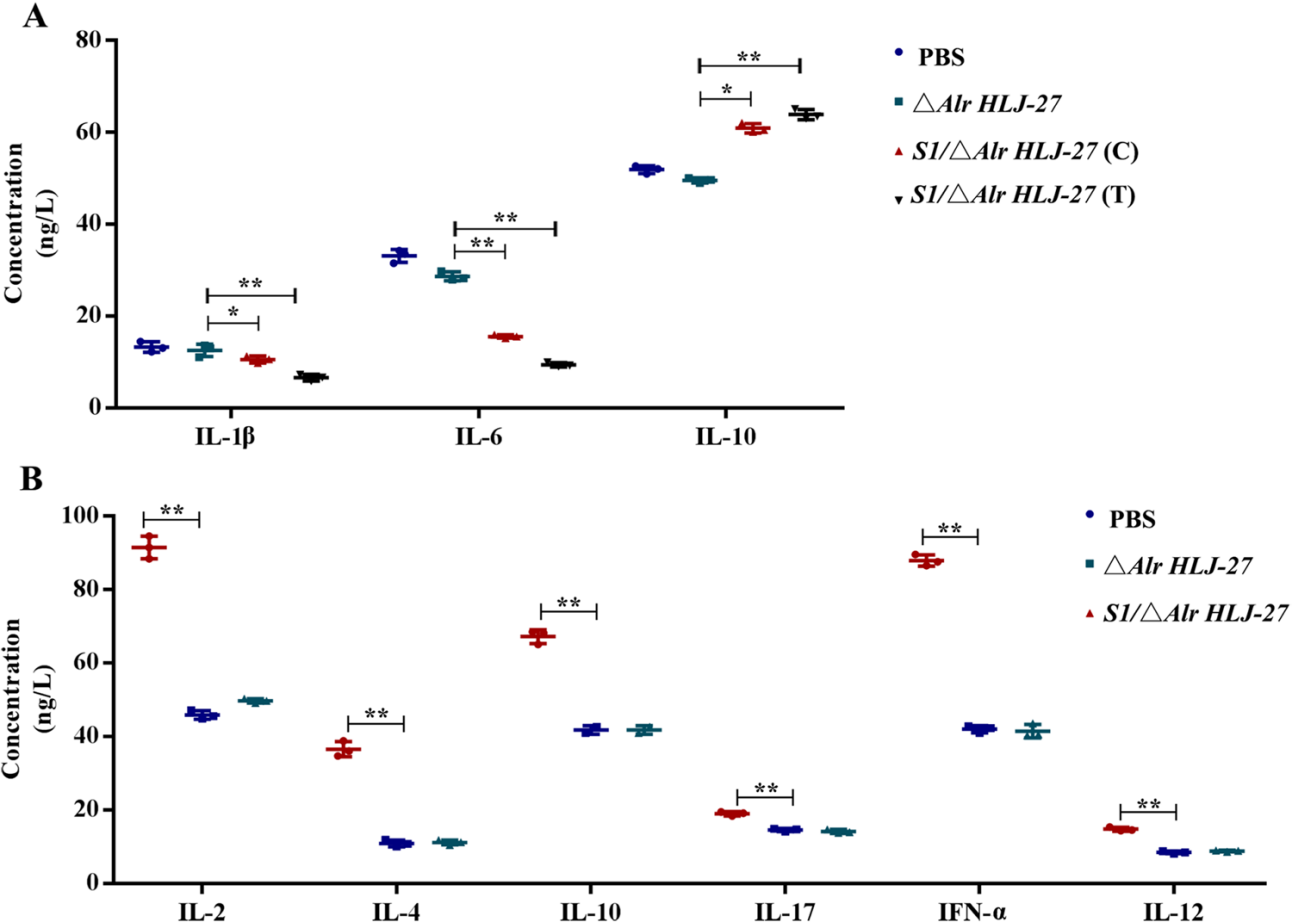
Detection of the levels of serum cytokines (piglets: *n* = 3 per group). The cytokines pre-challenge (**B**) and post-challenge (**A**) in piglets with oral immunization the mutant strain *S1/*△*Alr HLJ-27* were analyzed. *C* represents the challenge experiments groups, and *T* represents the piglets, housed in the same cage with the challenge groups. Bars represent the mean ± standard error value of each group (**p < 0.01 compared to the control groups PBS and △*Alr HLJ-27*)

During the oral immunization of piglets, serum samples were collected from the anterior vena cava on day 6 after the initial immunization and cytokine levels were examined using ELISA kits. Compared with the △*Alr HLJ-27* and PBS groups, the IL-2, IL-4, IL-10, IL-12, IL-17, and IFN-γ levels of the mutant *S1/*△Alr *HLJ-27* group were significantly increased, consistent with the experimental results found for mice (Fig. 8B).

In the challenge test, weight changes and the mental state of piglets were monitored. In the PBS and △*Alr HLJ-27* challenge groups, piglets developed a marked watery diarrhea, along with mental depression and extreme emaciation. In the *S1/*△*Alr HLJ-27* challenge group, piglets manifested mild diarrhea, slight weight loss, and good mental state. In the *S1*/△*Alr HLJ-27* co-cage infected group, a few piglets showed soft stools, good mental state, and normal appetite. An autopsy was performed immediately as the piglets were on the verge of death, and serum was collected simultaneously to detect cytokines related to the development of an inflammatory response (Fig. 8A). In this study, we examined the cytokine levels associated with the pro-inflammatory factors, IL-6, IL-1β, and the anti-inflammatory factor IL-10. After the piglets were challenged with PEDV, the IL-6 and IL-1β levels were significantly decreased in both the challenge and cohabitation infection groups with oral administration of the mutant strain *S1/*△*Alr HLJ-27*. Furthermore, the levels in the cohabitation infection group were lower than those in the challenge group. The IL-10 level showed a noticeable rise in both the challenge group and the cohabitation infection group with the strain *S1/*△*Alr HLJ-27*, and the cohabitation infection group exhibited higher levels than the challenge group. These results reveal that oral administration of the mutant strain *S1/*△*Alr HLJ-27* relieved the inflammatory response and elicited PEDV infection to some extent.

### Determination of the viral load of intestinal tissue

**Fig. 9:**
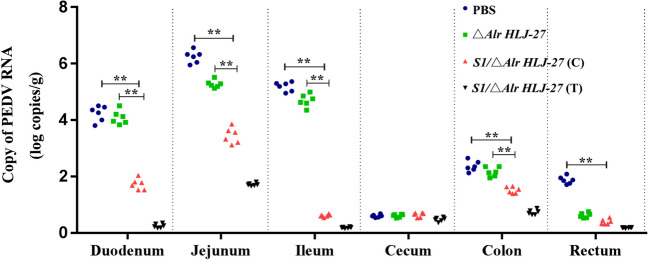
Detection of viral load in different intestinal tissue in piglets (*n* = 3 per group) after challenge with PEDV. The piglets were administered infection with 1 mL of PEDV intestines crude extract at 6 days post primary immunization. *C* represents the challenge experiments groups, and *T* represents the piglets, housed in the same cage with the challenge groups. With the death of the piglets, necropsy was performed immediately to collect each intestinal fragment for virus detection. Bars represent the mean ± standard error value of each group (**p < 0.05, ** p < 0.01 compared to the control groups PBS and △*Alr HLJ-27*)

To detect the viral load in each intestinal tissue of piglets challenged with PEDV, the tissue was ground to extract RNA, reverse-transcribed to cDNA, and tested using RT-PCR. As shown in Fig. 9, compared to the △*Alr HLJ-27* and PBS groups, the viral load in the intestinal tissues of the challenge group and the cohabitation infection group with the orally administered *S1/*△*Alr HLJ-27* was markedly decreased, and the viral load in the cohabitation infection group was lower than that in the challenge group. These results revealed that oral administration of strain *S1/*△*Alr HLJ-27* had a protective effect on piglets.

### Gross Lesion and Histopathological Examinations

**Fig. 10:**
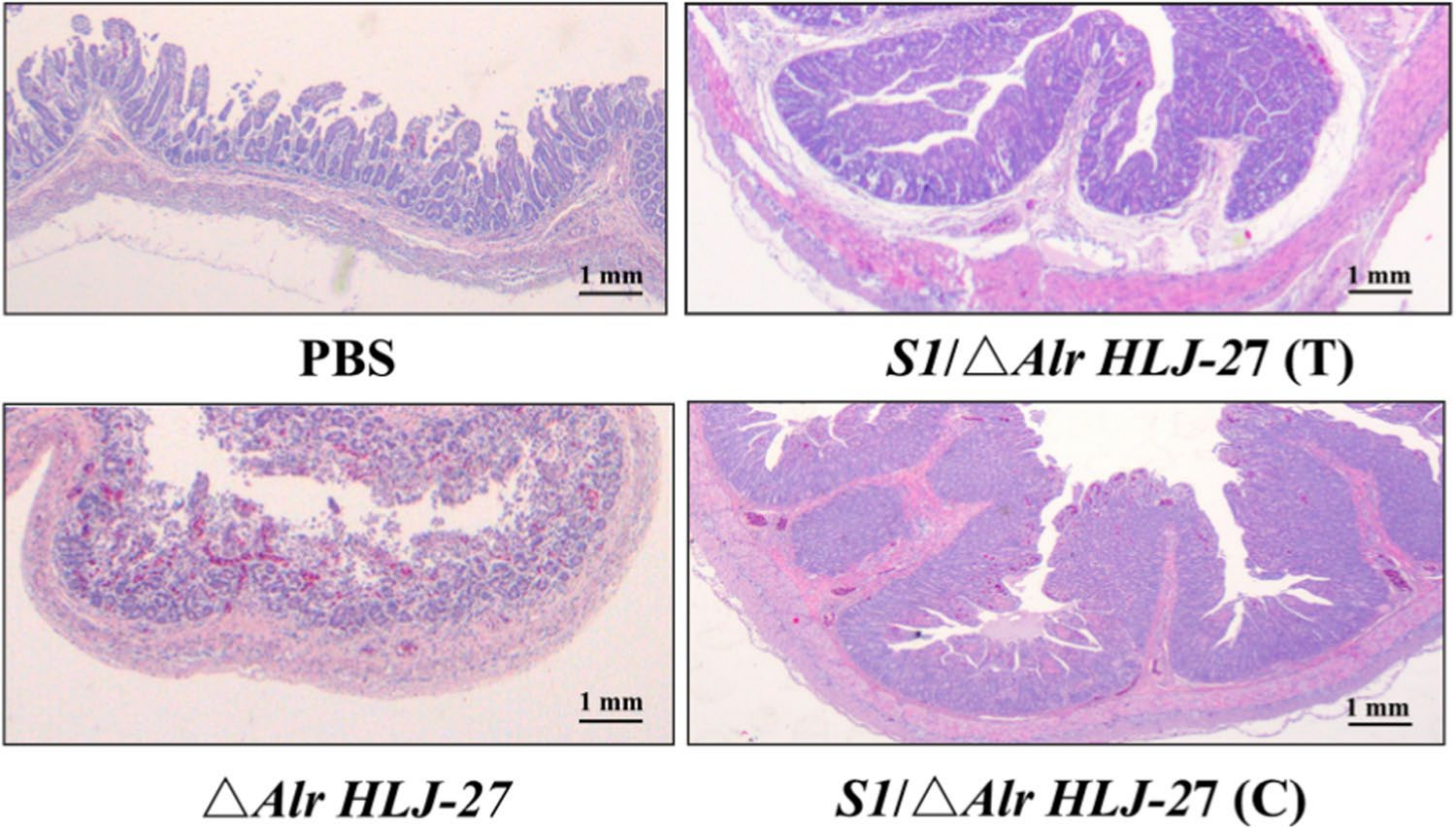
Histopathological analysis of the strain *S1*/△*Alr HLJ-27* protective effect on immunized piglets. Histopathological examination of H&E-stained small intestinal tissues. The tissues were acquired from the groups administered PBS, △*Alr HLJ-27* and *S1*/△*Alr HLJ-27*, post-challenge with 1 mL of PEDV intestines crude extract (RNA copy number of 1.0 × 10^6^). “*C*” represents the *S1*/△*Alr HLJ-27* challenge experiments groups, and “*T*” represents the *S1*/△*Alr HLJ-27* piglets, housed in the same cage with the challenge groups. With the death of the piglets, necropsy was performed immediately to collect each intestinal for analysis. Original magnifications: 1 mm

Small intestinal tissues from different treatment groups were collected for histopathological analysis (Fig. 10). Lesion on small tissues in PBS and △*Alr HLJ-27* groups was more serious than that in *S1/*△*Alr HLJ-27* groups, either the challenge experiments group or the co-cage infection group. Samples from the PBS group showed that the small intestinal villi were highly atrophic, necrotic, and even disaggregated and shed; the number of villi was significantly reduced; and some mucosal epithelial cells were shed. In △*Alr HLJ-27* group, the villi of the small intestine were significantly atrophic, and the mucous epithelial cells degenerated and shed. Compared with PBS and △*Alr HLJ-27* groups, the degree of villus atrophy in the small intestine of *S1/*△*Alr HLJ-27* group was not obvious. The small intestinal villi were more intact in the cocage infected group than in the direct infected group.

## Discussion

LAB are widely used as live vaccine carriers due to their safety, adjuvant properties, and weak immunogenicity (Xu et al. 2019). Traditional vaccines include inactivated vaccines and attenuated vaccines, but they all have certain defects. The immune response induced by intramuscular injection of inactivated vaccine takes a long time and could not effectively stimulate the mucosal immune response dominated by sIgA (Kim et al. 2023). The attenuated vaccine could induce the production of specific antibodies quickly and at high levels, yet there is a potential risk of reversion and enhancement of virulence (Teng et al. 2022). Researchers are committed to develop LAB oral vaccines, using plasmids to express heterologous proteins (Yang et al. 2018, Maqsood et al. 2018). Plasmid expression systems are often used to deliver foreign genes and antibiotics as selective markers, but this approach can cause issues such as the emergence of residual resistance genes and genetic instability (Douglas and Klaenhammer 2011), so to attempt to integrate foreign genes into the LAB genome to overcome these limitations.

Gene integration in LAB genome generally utilizes a double crossover recombination system, but the efficiency is low and the operation is complex (Goh et al. 2009, Hols et al. 1994). The development of the CRISPR-Cas9 system has revolutionized gene editing in LAB, enabling convenient and rapid expression of foreign genes within the genome (Wang et al. 2021). In this study, the mutant strain *S1/*△*Alr HLJ-27* was constructed using a thermosensitive plasmid pLCNICK as the skeleton with the CRISPR-NCas9 system. This system performed a point mutation procedure on the Ruv C or HNH nuclease domains of Cas9 (Gasiunas et al. 2012). The mutated Cas9 protein (NCas9) cleaves only one strand of DNA, and a single incision is made without causing double-strand breaks in the DNA (Wu et al. 2022). Song et al. successfully employed the CRISPR-NCas9 system to perform four-gene knockout and insert the foreign gene EGFP into the genome with an efficiency range of 25–62% (Song et al. 2017). In our study, the S1 gene was integrated into the genome of △*Alr HLJ-27* at the thyA site with an efficiency of 50% (the data was not shown), consistent with previous findings. Oh et al. showed that combining the CRISPR-Cas9 system with ssDNA recombination enhanced the efficiency of editing *Lactobacillus reuteri* to 100% (Oh and van Pijkeren  2014). Similarly, Guo et al. integrated optimized ssDNA recombination with the CRISPR/Cas9 system and successfully achieved sequential point mutation targeting the upp and galK gene loci, improving the gene-editing efficiency to 75% (Guo et al. 2019). These results provide new insights into gene editing in LAB.

In this study, thyA, encoding thymidylate synthase, was selected as the target site for genome integration. Lothar Steidler et al. substituted the IL-10 gene for thyA in the *Lactococcus lactis* genome and developed a validated therapy for chronic colon disease, which showed promising initial results (Steidler and Rottiers 2006). Zhou et al. successfully expressed bovine lactoferrin peptide with thyA site, and the results revealed that it was well resistant to pathogenic *Escherichia coli* and *Staphylococcus aureus* infection (Zhou et al. 2018). In the present study, we introduced a combination of P776, the promoter of the pyruvate hydratase gene, along with S1, to replace the thyA locus. Yin et al. demonstrated through microarray experiment analysis that the pyruvate hydratase gene could be constitutively and efficiently expressed in the genome of *L. casei*, and this gene shares a high homology of up to 90% with many other LAB species. Researchers further utilized this site to integrate the VP4 gene of rotavirus into the LAB genome, with the promoter initiating the transcription of foreign genes, indicating that heterologous proteins could be expressed stably and efficiently in the host bacteria (Yin et al. 2016). The expression of the S1 gene was successfully detected by western blot analysis and proved the exogenous protein could be stably inherited within 20 generations, further validating the operability of the above findings.

Subsequently, the plasmid residues of the mutant strain were tested by extracting plasmids of the 1st and 20th generation strains, and PCR amplification results showed that the plasmid was either undetectable or completely eliminated, consistent with the study by Song et al. (Song et al. 2014a, 2014b), precluding the negative influence of antibiotics as screening markers. The strain △*Alr HLJ-27*, which was developed in our laboratory to restore growth through d-alanine supplementation (Li et al. 2022), was selected as the host bacteria. The mutant strain *S1/*△*Alr HLJ-27*, generated in this study, retained this characteristic. There was a great significance for the artificial control of strains and the protection of intellectual property rights. The construction of △*Alr HLJ-27* was based on the original wild-type strain *HLJ-27*, isolated from the intestine of large landrace piglets, as previous studies have shown that *Lactobacillus* isolated from the intestine of piglets has stronger colonization ability than *Lactobacillus* from other sources (Liu et al. 2014).

PEDV primarily destroys the intestinal epithelial tissue of piglets, highlighting the crucial role of mucosal immune SIgA in their defense to viral invasion. SIgA acts as a vital barrier against pathogen invasion in the gastrointestinal tract and neutralizes virions in the gut (Wang et al. 2018). Several studies have investigated the preparation of oral vaccines against PEDV. Wang et al. devised a mucosal DC-targeted oral vaccine using *L. casei* as the host to express DCpep and the COE gene of PEDV. Mice orally immunized with this vaccine exhibited effective induction of SIgA and IgG production (Wang et al. 2017). Li et al. applied alanine racemase-deficient *L. casei W56* as the host strain and a complementary plasmid as a vector to express the neutralizing antigen COE of PEDV. After the oral immunization of mice, both SIgA and IgG levels increased significantly (Li et al. 2021). In this study, the protective antigen S1 gene of PEDV was inserted into the genome of the strain △*Alr HLJ-27* to construct the mutant strain *S1/*△*Alr HLJ-27*. Mice and piglets were orally immunized with the strain to evaluate its immune effect.

In the mouse immunization experiment, we detected SIgA levels in tears, genital tract washing fluid, feces, and intestinal mucus. The results showed that the levels of SIgA in each index increased significantly, indicating that the mutant strain *S1/*△*Alr HLJ-27* in mice could elicit an effective mucosal immune response. Jiang et al. used *L. casei W56* to present H antigen of canine distemper virus, then orally immunized mice to induce effective mucosal immune responses (Jiang et al. 2019). A piglet immunization experiment was performed, and we observed a significant increase in SIgA level in anal and nasal swabs of piglets immunized with the mutant strain *S1/*△*Alr HLJ-27*, as compared with the groups administered PBS and △*Alr HLJ-27*. The immune results in piglets were consistent with those observed in mice, indicating that the strain *S1/*△*Alr HLJ-27* could elicit mucosal immune responses in animals. SIgA antibody production was also detected in the experimental results of the nasal and anal swabs of Jiang and Hou et al. (Hou et al. 2018; Jiang et al. 2016). The mouse immune experiment served as a preliminary experiment for the development of the PEDV oral vaccine and laid the foundation for subsequent immune experiments on piglets. However, SIgA antibodies represent the primary defensive mechanism of mucosal immunity, while serum IgG level also plays a role in preventing pathogen invasion of the intestinal mucosa and systemic transmission, replenishing the mucosal protective mechanisms supplied by SIgA (Wells and Mercenier 2008). In the present study, a significant and sustained anti-PEDV IgG response was observed in *S1/*△*Alr HLJ-27* mice, which peaked at 6 weeks post-immunization. Similar results were obtained in piglets, with a significant increase in serum IgG levels after oral immunization with the *S1/*△*Alr HLJ-27* strain. Oral LAB vaccines can elicit antigen-specific humoral immune responses in animal models. Yu et al. demonstrated that the LAB oral vaccine could also induce humoral immunity. Recombinant *L. casei* was constructed by expressing protective antigens such as PEDV, TGEV, and eGFP as reporter genes. Serum IgG levels in mice increased significantly after oral immunization with the recombinant strain (Yu et al. 2017). The production of neutralizing antibodies is an important indicator of the ability of a LAB vaccine to provide immune protection. Neutralizing antibody levels in the serum of immunized mice and piglets were evaluated on days 42 and 6. Compared to the control groups, oral administration of the S1/△*Alr HLJ-27* strain led to a significant rise in neutralizing antibody levels, indicating that the engineered strain was capable of inducing the production of neutralizing antibodies. Yin et al. successfully detected the presence of neutralizing antibodies in *L. casei* oral vaccine immunization experiments using mice as a model animal (Yin et al. 2016). Thereby providing effective protection for mice and piglets.

Analyzing secreted cytokines is vital for assessing the capacity of a genetically engineered vaccine to stimulate Th1 or Th2 responses (Rasquinha et al. 2021). IL-12 facilitates the differentiation of CD4^+^ T helper (Th0) cells into Th1 and Th2 cells, which produce cytokines and participate in immune regulatory functions, including the generation of neutralizing antibodies and the modulation of cellular immune responses (Mailliard et al. 2019). Cytokines secreted by Th1 cells, such as IL-2 and IFN-γ, are closely associated with cellular immune responses, whereas cytokines produced by Th2 cells, such as IL-4 and IL-10, are relevant to humoral immune responses (Nan et al. 2007). During this experiment, statistically significant changes were observed in the IL-2, IL-4, IL-10, IL-12, IL-17, and IFN-γ cytokines, indicating that the strain *S1/*△*Alr HLJ-27* successfully induced Th1, Th2, and Th17 cellular immune responses in orally immunized mice and piglets. Xiao et al. utilized the strain *L. casei* deficient in the upp gene to express the PEDV S1 protein, orally immunized mice, and measured cytokine levels. The data revealed the successful induction of Th1, Th2, and Th17 cell immune responses (Xiao et al. 2022). Zhang et al. developed the alanine racemase-deficient *L. casei* complementary system to express the VP4 gene of porcine rotavirus. The obtained results were consistent with the research of Xiao (Zhang et al. 2022). Recently, the activation of SIgA secretion to combat invasion by pathogenic microorganisms was demonstrated to be a T-cell-dependent immune response (Fagarasan et al. 2010). In this study, the S1 gene expressed by △*Alr HLJ-27* was able to elicit high levels of SIgA secretion and both Thl- and Th2-type immune responses simultaneously, confirming the critical role of efficient T-cell–mediated immunity in SIgA production.

PEDV primarily infects the intestinal tract of piglets (Zhang et al. 2020); therefore, the development of an oral vaccine would play a key role in combating viral invasion. In previous studies, a recombinant *L. casei W56* strain expressing a dendritic cell-targeting peptide fused with the PEDV COE antigen was developed to assess its immune protective effect in piglets. The genetically engineered strain could provide protection at a rate of 60% (Hou et al. 2018). To analyze the protective effect of the immune response induced by oral immunization with *S1/*△*Alr HLJ-27* in piglets, we performed a challenge experiment. The piglets administered *S1/*△*Alr HLJ-27* were divided into two groups: one group was carried out the challenge test, and the other was raised in the same cage. Quantitative reverse-transcription PCR was used to detect the copy number of PEDV RNA in each group of small intestinal tissues. Compared to the control groups, the immunized group showed a significant decrease in PEDV copy number. The copy number of PEDV in the cohabitation infection group of piglets immunized with the *S1/*△*Alr HLJ-27* strain was the lowest among all groups. Histopathological results showed that compared with PBS and △*Alr HLJ-27* groups, the extent of PEDV disruption to the small intestine was significantly reduced, and the villi were relatively intact. Hou and Wang et al. also showed that oral administration of recombinant lactobacilli expressing heterologous proteins could effectively protect the small intestinal villi from viral destruction (Hou et al. 2018, Wang et al. 2019). These results indicate the ability of specific SIgA and IgG antibodies to neutralize PEDV virus particles and contribute to immune protection in piglets to some extent.

During viral invasion, PEDV inflicts severe pathological damage on small intestine tissue, and its progression is closely related to the inflammatory response (Chen et al. 2020). Abnormal expression of proinflammatory factors in the gut, with abnormal elevation, results in a cascade of inflammation, tissue damage, and increased permeability of the intestinal barrier (Wu et al. 2016). In this study, we evaluated the levels of IL-1β, IL-6, and IL-10 in the serum of piglets in a challenge experiment. IL-1β and IL-6 served as typical inflammatory factors, while IL-10 represents the main anti-inflammatory factor in the intestines (Trapecar et al. 2014; Martin and Ales 2014, Roselli et al. 2016). Song et al. used mice as a model of acute colitis to study the therapeutic effect of bovine antimicrobial peptides; they measured the indexes of IL-1β, IL-6, and IL-10 related to the inflammatory response (Song et al. 2019). Xie et al. also analyzed the corresponding inflammatory factor indexes IL-1β, IL-12, IL-6, TNF-α, and IL-10 in the process of recombinant *Lactobacillus reuteri* against pathogenic *Escherichia coli* invasion using piglets as a model (Wang et al. 2021; Xie et al. 2021). Our findings showed that the levels of IL-1β and IL-6 in the serum of piglets significantly increased following PEDV challenge. After oral administration of genetically engineered strains to piglets, however, this secretion trend was ameliorated, and the level of IL-10 increased, suggesting a positive effect on the inflammatory status of the piglets. In piglets treated with the orally administered engineered strain, the levels of IL-1β, IL-6, and IL-10 in the co-infection group were lower than those in the challenge group, indicating that the inflammatory response caused by PEDV was milder in the co-infection group. This finding further supports the conclusion that recombinant bacteria provide immune protection for piglets.

To develop an oral vaccine against PEDV, we constructed a recombinant strain, *L. paracasei S1/*△*Alr HLJ-27*, using the CRISPR-NCas9 system. Our results indicate that *S1/*△*Alr HLJ-27* could effectively activate mucosal, humoral, and cellular immune responses in mice and piglets following oral administration providing certain degree of protection against PEDV infection in piglets.

## Data Availability

The data that support the findings of this study are available from the corresponding author, Xiaona Wang, upon reasonable request.
